# Experimental characterization of Spherical Bragg Resonators for electromagnetic emission engineering at microwave frequencies

**DOI:** 10.1038/s41598-023-47059-y

**Published:** 2023-11-22

**Authors:** Yalina García-Puente, Jean-Jacques Laurin, Raman Kashyap

**Affiliations:** 1 Department of Engineering Physics, Poly-Grames Research Centre, Polytechnique Montreal, 2900 Édouard-Montpetit, Montreal, QC H3T 1J4 Canada; 2 Department of Electrical Engineering, Poly-Grames Research Centre, Polytechnique Montreal, 2900 Édouard-Montpetit, Montreal, QC H3T 1J4 Canada

**Keywords:** Photonic crystals, Microwave photonics, Photonic devices

## Abstract

This work reports experimental investigation and numerical validation of millimeter-sized Spherical Bragg Resonators (SBRs) fabricated using 3D printing technology. The frequency dependencies of the reflection and transmission coefficients were analyzed, and eigenfrequency values were calculated to examine the density of photonic states in air/PLA-polylactide SBRs, showing the appearance of an eigenmode and an increase in the local density of states in the core of a defect cavity. A decay rate enhancement of ${\sim 10}^{2}$ was obtained for a dipole placed in the core of the defect SBR. The study also investigated the influence of the source position on the resonator's electromagnetic wave energy. Scattering efficiencies up to order twelve of the multipole electric and magnetic contribution in a 10-layer SBR were calculated to validate the presence of the resonant modes observed in the scattering measurements performed for parallel and perpendicular polarizations. The results demonstrate that SBRs can act as omnidirectional cavities to enhance or inhibit spontaneous emission processes by modifying the density of electromagnetic states compared to free space. This finding highlights the potential of SBRs engineering spontaneous electromagnetic emission processes in various applications, including dielectric nanoantennas, optoelectronics devices, and quantum information across the entire electromagnetic spectrum.

## Introduction

Over the last few years, the interest in photonic structures with three-dimensional (3D) band gaps created by periodic scattering^1–3^ at optical and longer wavelengths has increased drastically. Yablonovitch^4^ proposed a 3D collection of spherical holes within a dielectric to attempt creating a photonic band gap. Although the spheres were resonant cavities, they failed to exhibit a band gap, as described in^4^. Meanwhile, hollow core photonic crystal fibers have been extensively used^5^, or other “onion skin’’ high contrast optical fiber has also found use in high power transmission of 10-micron radiation^6^. In the cylindrical geometry, “onion skin” concentric cylindrical layers waveguides have also been demonstrated for mode propagation direction with transverse resonant confinement^7^. The other type of 3D photonic crystals is radial periodic structures, developed in spherical coordinates in the form of spheres with a mutual arrangement of the centers of the multilayer sphere^8–10^. When the optical thickness of the layers is about a quarter wavelength, the multilayer sphere of alternately high and low refractive index can confine photons within its core. This type of structure is known as Spherical Bragg Resonator (SBR)^11,12^. The unique property of these SBRs is that the 3D wave function is enclosed in the structure, which could be used to control spontaneous emission^13,14^, to develop a single-photon source^15^ or threshold-less lasers^16,17^.

Previous studies have explored the light scattering of SBRs using various theoretical methods^9,18,19^, such as the plane-wave^20^ and finite-difference time-domain methods^21^. More recently, the development of a numerical mode-solving technique called spherical Bessel-Legendre-Fourier space has allowed resonance state frequencies and field profiles to be obtained for any lossy material or dielectric structure with arbitrary characteristics^22^. However, the recursive transfer matrix method developed by Moroz^19^ has become the most widely used matrix formulation for modeling multilayer spheres. This method offers an analytical solution for the radiative decay rate of a radiating dipole located at any position within a multilayer sphere, its scattering efficiencies, and electromagnetic energy distribution^19,23,24^. The accuracy of this method for modeling different spherical multilayer plasmonic structures has been demonstrated in previous works^23,25,26^. In particular, we have already used the algorithm to study the engineering quantum dipolar electric or magnetic transitions in the presence of SBR^27,28^. Moreover, we have explored the potential of SBR to improve the performance of dielectric nanoantennas and for optical amplification and lasing^27^.

Currently, the production of SBR is mainly based on conventional techniques requiring clean-room technology, including electron-beam deposition of ZnS/Na_3_AlF_6_^29^, multistage emulsion polymerization of polystyrene/poly(trifluoroethyl methacrylate)^30^, sol–gel and homogeneous precipitation techniques to prepare SiO_2_ and Y_2_O_3_ shells, respectively^31^, etching and plasma-enhanced combined with chemical-vapour deposition^16,32,33^. Medvedev et al.^32,33^ fabricated two types of SBR for near-infrared applications. These comprise alternating quarter-wave layers of amorphous hydrogenated silicon carbide (*a*-Si_1-x_C_x_ : H), and amorphous silicon oxide (*a*-SiO_2_) placed over erbium ions doped and undoped glass microspheres^32,33^. Nevertheless, there is no fully established technique to replicate the deposition of thin layers with homogeneous chemical composition onto a microscopic spherical particle. This lack of a reliable fabrication method has limited the exhaustive experimental characterization of SBRs and their potential applications that have been theoretically envisioned^27^.

Dielectric resonators have played an indispensable role in microwave technology due to their renowned ability to provide exceptionally high spectral purity and precise frequency stability^34,35^. They are essential components in various applications, including microwave sources and filters. The extensive literature on spherical and hollow core resonators underscores their significance, given their capacity to support whispering-gallery modes characterized by extraordinarily high-quality factors (Q-factors)^36–38^. Spherical Bragg Resonators, however, represent a novel development that has opened the door to achieving Q-factors in the microwave domain that can attain or even exceed one million^11,39,40^. Traditionally, dielectric resonators have been crafted from a diverse range of ceramics and materials. For instance, constructing a Teflon-free space resonator demonstrated a Q-factor of 22,000, primarily limited by the dielectric losses of Teflon^41^. It is worth highlighting that the use of lower-loss materials, such as sapphire, and the strategic incorporation of additional layers have made it possible to achieve higher Q-factors, as documented in the existing literature^40,41^. Over the past few decades, 3D printing has attracted attention as a versatile and low-cost manufacturing tool for a wide range of applications^42,43^. The spatial resolution of the 3D printing allows the fabrication of the photonics structure in the microwave frequency range^44–46^.

This paper focuses on an experimental investigation and numerical validation of the performance of Spherical Bragg Resonators (SBRs) produced using 3D printing technology. In our study, we examine the dependence of the reflection and transmission coefficients. Furthermore, we calculate the eigenfrequency values to analyze the density of photonic states within the SBRs. We also explore the source position's impact on the resonator's electromagnetic wave energy. Additionally, we determine the scattering efficiencies of the multipole electric and magnetic contribution in a 10-layer SBR up to order twelve ($l=12$) and compare them with the scattering measurements for parallel and perpendicular polarizations. The findings demonstrate that SBRs can function as a cavity, modifying the density of electromagnetic states compared to free space and enhancing or inhibiting spontaneous emission processes. The manuscript underlines the potential of SBRs for precisely controlling spontaneous electromagnetic emission processes in various applications, including dielectric nanoantennas, optoelectronics devices, and quantum information systems, covering the entire electromagnetic spectrum. 3D printing technology in SBR fabrication offers a scalable and accessible method for producing these resonators, further expanding their potential for practical applications.

## Results and discussion

**Figure 1:**
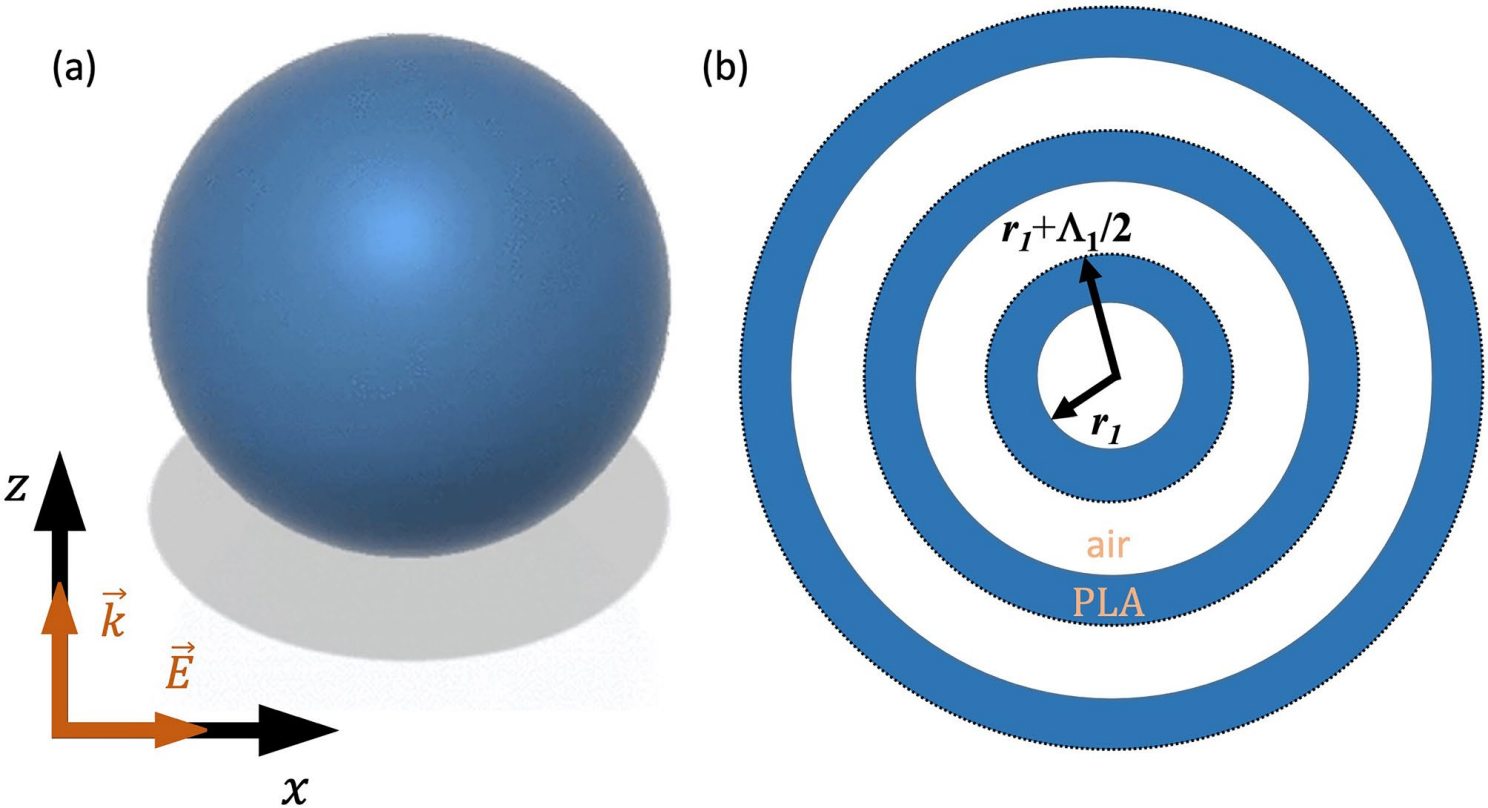
(**a**) Schematic of a Spherical Bragg Resonator excited by a plane wave. (**b**) Cross section of a Spherical Bragg Resonator ${r}_{i}$ represents the radius of the spheroid and ${\Lambda }_{j}$ is some design factor.

Figure 1a shows the schematic representation of the investigated Spherical Bragg Resonator (SBR). This SBR consists of an air spherical core, surrounded by concentric dielectric PLA shells. The multilayered spheres is illuminated by a planar electromagnetic wave and the surrounding medium is air. Figure 1b illustrates N-concentric spheroids with each spheroid spaced by a radius increment, ${r}_{i+1} = {r}_{i} + {\Lambda }_{j}/2$, where ${r}_{i+1}$ is the radius of the next outer spheroid, ${\Lambda }_{j}$ is one design factor, i.e. wavelength selected for each spheroid, with $0\le i\le N$ and $1\le j\le N$. The phase-matching condition for resonance is given by:

1 $$2{n}_{eff}{\Lambda }_{p}={\lambda }_{B}$$

where, ${n}_{eff}$ is the effective refractive index, and ${\Lambda }_{p}=2\left({r}_{i+1}-{r}_{i}\right)$. Here we have assumed that there is no dependence of the propagation constant with the azimuthal angles, $\theta$ and $\varphi$.

In our study, the thickness of each shell $\left({r}_{i+1}-{r}_{i}\right)$ is fixed at about one-quarter of a wavelength (${\lambda }_{B}/4{n}_{i}$), which results in constructive interference of waves reflected from each interface, creating a frequency range known as the band gap, where electromagnetic waves cannot propagate outward from the resonator. The SBR has a spherical symmetry in the radial direction of light propagation, forming an omnidirectional band gap. The wavelength at which the SBR acts as an omnidirectional Bragg reflector is defined as the center of the band gap, denoted as ${\lambda }_{B}$.

### Reflectance spectra and local density of states

**Figure 2:**
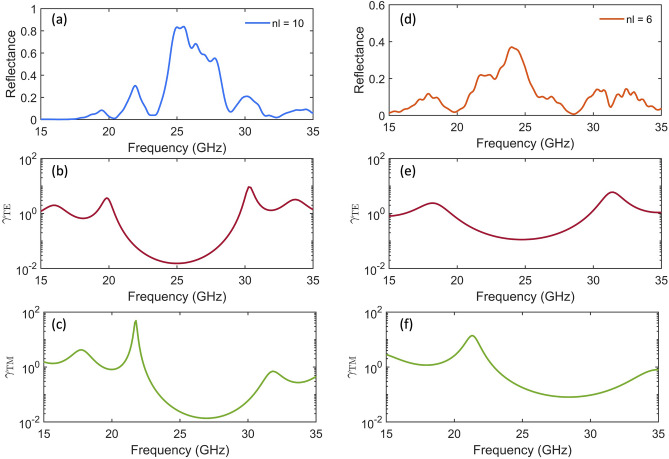
Measured reflectance spectrum of SBRs with a different number of layers, (**a**) ${n}_{l}=10$ and (**d**) ${n}_{l}=6$, at the core of the structure. Calculated dipolar spontaneous emission decay rate $\gamma$ for the transversal electric (**b**) and (**e**) and transversal magnetic (**c**) and (**f**) polarizations. In both geometries, the SBR band gap's center is fixed at ${f}_{B}=25 \; \mathrm{GHz},$ and the core radius is ${r}_{core}={\lambda }_{B}/4{n}_{air}$.

Photonic bandgap in 3D-printed PLA-polylactide (PLA)^47^ SBRs were studied by placing a dipole antenna at the center of the 3D photonic crystal. The methodology section includes a schematic representation of the measurement setup considered in this work. Reflectance measurements shown in Fig. 2a and d were taken for air/PLA Spherical Bragg Resonators with varying layer numbers, ${n}_{l}=10$ and ${n}_{l}=6$. These resonators have Mie size parameters of $2\pi R/{\lambda }_{B}=14.16$ and $9.12$, and an air core radius of ${r}_{core}={\lambda }_{B}/4{n}_{core}$ with Bragg frequency ${f}_{B}=c/{\lambda }_{B}=25 \; \mathrm{GHz}$, where *c* is the speed of the light and $R$ the outermost radio. In both cases, we obtained frequency ranges where the reflectance is maximal, corresponding to the photonic stopband. The results revealed that the periodic layering of alternating air and PLA materials in the SBR led to partial reflection of the incident electromagnetic wave at each spherical boundary. The constructive interference of the reflected waves at a frequency approximately four times the optical thickness of the concentric spherical shells resulted in the concentric spherical layers acting as mirrors. Therefore, the increase in coatings resulted in higher maximum reflectance. This observation can be attributed to the fact that more air/PLA spherical layers led to more significant constructive interference of the reflected waves, which enhanced the reflectance. Due to the 3D printer constraints, our study was limited to SBRs with ${n}_{l}=10$ and ${n}_{l}=6$. However, based on observations in the planar case^6^, it is expected that with an increase in the number of layers in the reflector, radiation within this wavelength range would be unable to propagate outward from the resonant structure.

Recent studies have explored various photonic structures, including *woodpile* and *inverse opal*, and demonstrated that the local density of photonic states increases within the eigenmodes of the cavity and at the boundaries of the photonic bandgap^48–50^. Our study focuses primarily on the decay rate of the spontaneous emission of an active atom placed at the core of the SBRs, which is proportional to the local density of states^51^. Specifically, we calculated a dipole's spontaneous emission decay rate for transverse electric (${\gamma }_{TE}$) and transverse magnetic (${\gamma }_{TM}$) polarizations.

To better understand the relationship between the photonic bandgap and dipolar emission behavior, we studied the spectral distribution of the radiative decay computed for SBRs with band gap's center fixed at ${f}_{B}=25 \; \mathrm{GHz},$ ${n}_{l}=10$ and ${n}_{l}=6$, and the core radius ${r}_{core}={\lambda }_{B}/4{n}_{air}$. The calculated dipolar spontaneous emission decay rate is shown in Fig. 2b and e for the $TE$ polarization and in Fig. 2c and f for the $TM$ polarization. As noted, the states' density decreases to zero within the photonic bandgap region, resulting in emission suppression. However, at the edges of the forbidden bandgap, the density of states increases substantially, leading to an increase in dipolar emission at the corresponding wavelengths. In the next section, we will analyze how defects in the resonator create eigenmodes in the core of the cavity and, consequently, an increase in the local density of states. Our results suggest that these structures permit control and enhance light-matter interactions in various applications, including sensing and energy harvesting.

### Geometrical and spatial dependence of the resonant modes

**Figure 3:**
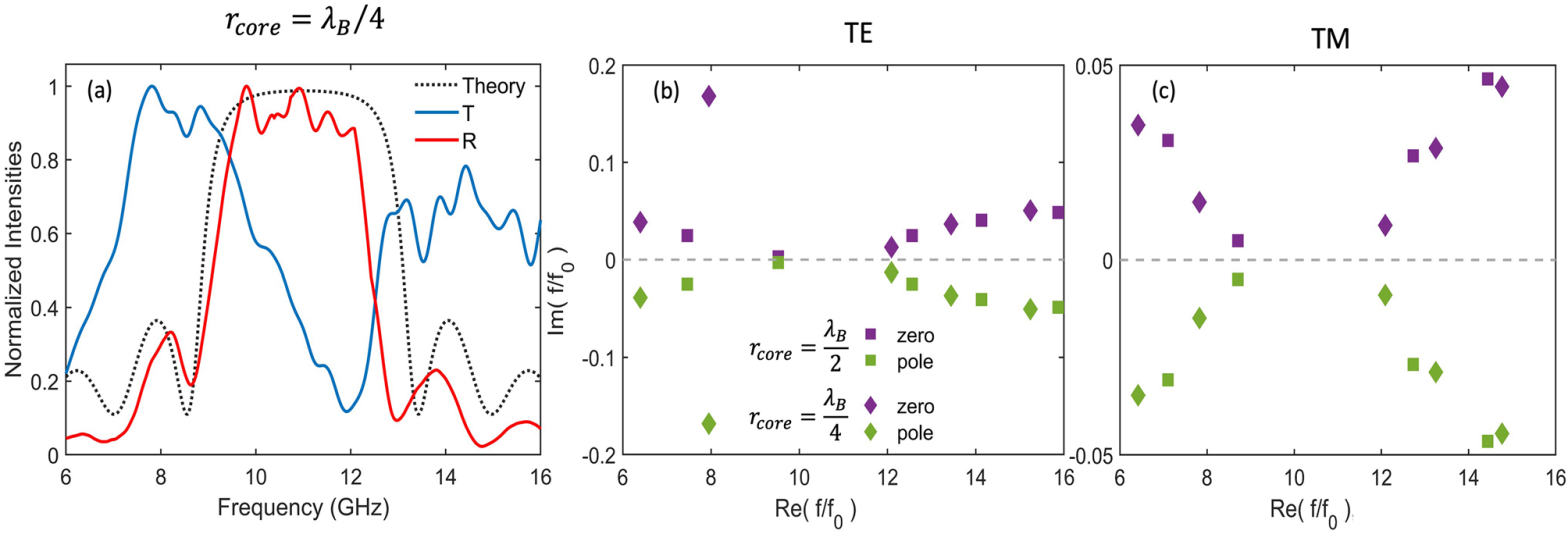
(**a**) Experimental and calculated reflectance spectrum at the center of an SBR with ${n}_{l}=10$ plotted in solid red and black dotted lines, respectively. Experimental transmitted power measured near the SBR surface was plotted in a solid blue line. All spectra are normalized to the maximum reflection coefficient. The SBR band gap's center is fixed at ${f}_{B}=11 \; \mathrm{GHz},$ and the core radius is ${r}_{core}={\lambda }_{B}/4{n}_{air}$. (**b**) Calculated poles and zeros of the SBR scattering matrix for two different core radii ${r}_{core}={\lambda }_{B}/2{n}_{air}$ and ${r}_{core}={\lambda }_{B}/4{n}_{air}$ represented as square and diamond symbols, respectively. The pole-zero map frequencies are normalized by ${{f}_{0}}=1 \; \mathrm{GHz}$.

Figure 3a displays the reflection ($R$) and transmission ($T$) spectrum measured for a 10-layer air/PLA SBR with a core radius of ${r}_{core}={\lambda }_{B}/4{n}_{air}$. The reflection spectrum was recorded from the center of the SBR, showing an omnidirectional bandgap centered at around 11 GHz. To measure the transmitted power, a horn antenna was placed near the surface of the SBR. The recorded reflectance spectrum reveals that the maximum reflectance position coincides with the minimum of the transmitted power recorded.

To confirm the validity of our experimental results, we conducted a numerical comparison of the measured reflection spectrum of the SBR (Fig. 3a, black dotted line). As has been demonstrated before^42^, the SBR reflection coefficient is influenced by the polarization of the spherical wave ($TE$ and $TM$) and its angular modal number ($l$) while being independent of the azimuthal modal number ($m$). Therefore, the omnidirectional photonic bandgap overlaps all partial bandgaps for the spherical waves of all polarizations with all angular modal numbers. Upon comparison of the experimental and numerical reflection spectra, we found that the maximum values of both spectra coincide. However, the measured bandgap width was narrower than the predicted values. Additionally, we observed some high reflection near 8 GHz, which can be attributed to the presence of spherical modes of higher order with different angular modal numbers when the core radius approaches the order of the wavelength used, as has been previously discussed by other researchers^18,52^.

As previously mentioned, the density of photonic states experiences a significant increase within the eigenmodes of the cavity and at the boundaries of the photonic bandgap. Different methods have been proposed to calculate the eigenmode of resonant structures^53,54^, in these works we applied a Weierstrass factorization^55^ to the scattering matrix elements of a 10-layer SBR. The notable advantage of this semi-analytical approach lies in its ability to yield the eigenfrequencies of our structure independently for each order rather than as a composite of the supported resonances. Importantly, it straightforwardly achieves this without requiring additional fitting parameters or coupling factors, as is often the case with coupled oscillator models^44^. In Fig. 3b and c, the calculated poles and zeros of the SBR scattering matrix for two different core radii (${\lambda }_{0}/2{n}_{air}$ and ${\lambda }_{0}/4{n}_{air}$) are represented by square and diamond symbols, respectively. The pole-zero map frequencies for the $TE$ and $TM$ polarizations are normalized by ${{f}_{0}}=1 \; \mathrm{GHz}$. By performing the factorization, we obtained the singularities of the SBRs. The zeros correspond to perfect absorption, while the poles represent the eigenfrequency.

The results show no eigenmodes in the bandgap region of the SBR with a core radius of ${r}_{core}={\lambda }_{B}/4{n}_{air}$. This agrees with suppressing the decay rate in the bandgap region previously observed in Fig. 1. However, a localized photonic state can be created within the bandgap in the presence of a disorder, like a localized defect in an SBR with a core radius of ${r}_{core}={\lambda }_{B}/2{n}_{air}$. For this case, the SBR acts as a defect optical cavity in which the surrounding spherical layers act as a mirror in all directions. These have been the primary research focus in photonic crystal emission modification^13,56^. For instance, we have previously proposed a defect Er^3+^ core doped Si/SiO_2_ SBR for optical amplification and lasing^27^.

**Figure 4:**
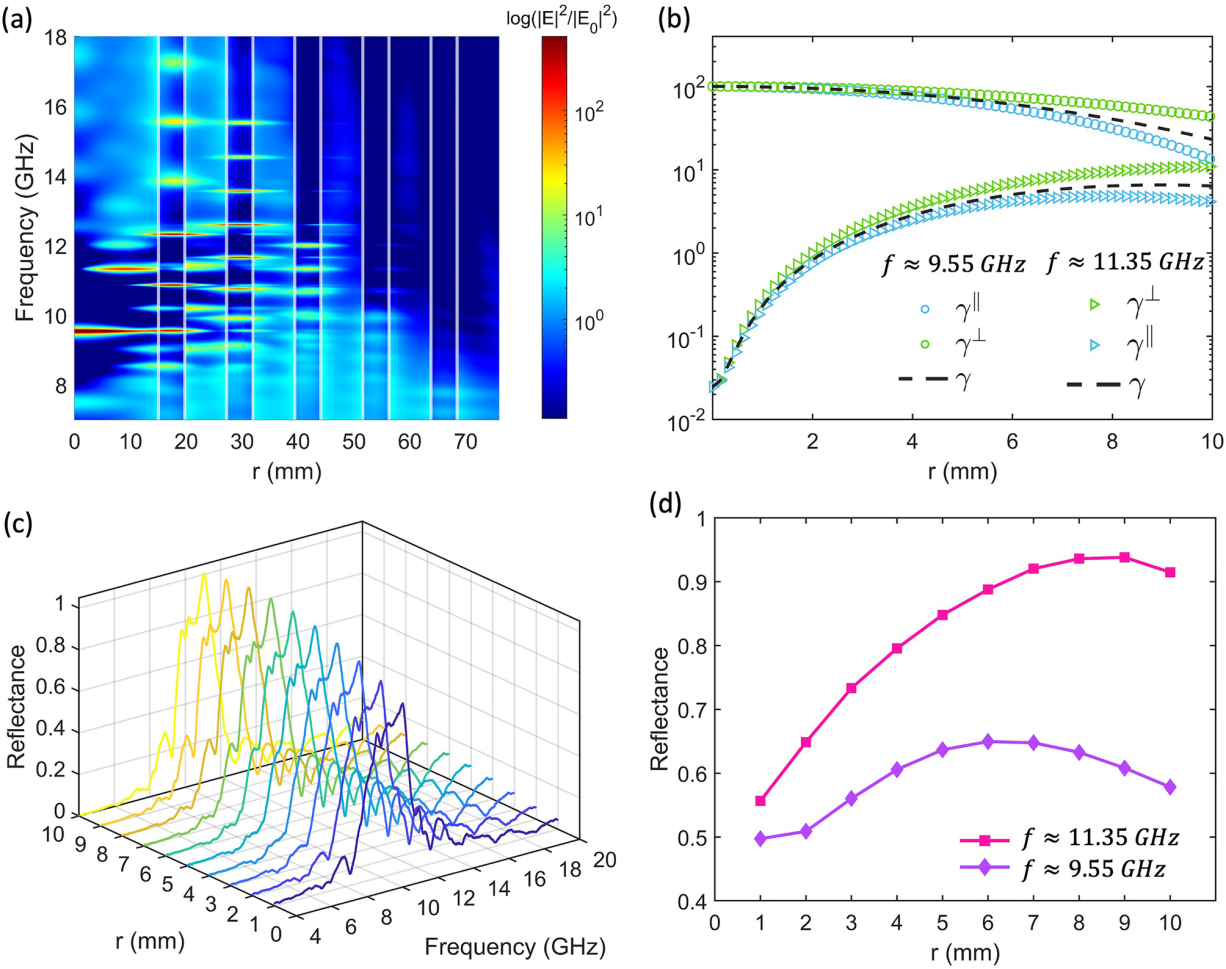
(**a**) Spatial distribution of the normalized electric field intensities of a 10-layer SBR with ${r}_{core}={\lambda }_{B}/2{n}_{air}$ as a function of the frequency. (**b**) Calculated total, perpendicular, and parallel decay rate components of a dipole at $f\approx 9.55 \; \mathrm{GHz}$, circular symbol, and $f\approx 11.35 \; \mathrm{GHz}$ triangular symbol as a function of the position along the SBR core. (**c**) Reflectance measurement at different points along the SBR core radius. (**d**) Reflectance measured at $f\approx 9.55 \; \mathrm{GHz}$ and $f\approx 11.35 \; \mathrm{GHz}$ at different points of the SBR core.

One essential part of our study of the SBR performance was to investigate the electromagnetic field localization inside the multilayer spheres. The radial distribution of the normalized electromagnetic energy for a defect 10-layer air/PLA SBR with ${r}_{core}={\lambda }_{B}/2{n}_{air}$ as a function of frequency is shown in Fig. 4a. As can be seen, the map depicts the location of the electric field within the different layers marked by white lines. A maximum value is observed at the defect mode of the resonator system ($f\approx 9.55 \; \mathrm{GHz}$) in concordance with the eigenfrequency value depicted in Fig. S1a. The maxima located in the core are of the Fabry–Pérot type and those located in the layers are of the whispering gallery mode type and are of higher order.

On the other hand, we calculated the total perpendicular and parallel decay rate components of a dipole at $f\approx 9.55 \; \mathrm{GHz}$ (circular symbol) and $f\approx 11.35 \; \mathrm{GHz}$ (triangular symbol) as a function of the position along the SBR core, as shown in Fig. 4b. These frequencies can be identified as maximum electromagnetic energy in the SBR core (see Fig. 4a) and correspond to two distinct eigenfrequencies, the independently estimated order or angular quantum numbers $l=1$ and $l=2$. Further details are in Fig. S1a and c. We observed that the decay rate for the dipole at the main resonant frequency or defect mode frequency ($f\approx 9.55 \; \mathrm{GHz}$) is several orders of magnitude higher than that of a dipole outside at the edge of the bandgap ($f\approx 11.35 \; \mathrm{GHz}$). However, the decay rates become closer as the dipole is moved away from the center. Additionally, we observed a divergence of the transverse and perpendicular components due to the breaking of the spherical symmetry when we moved out of the center of the core. The minor shift in the decay rate of the dipole at the principal resonant frequency is worth noting, which provides us with flexibility when designing practical devices.

Furthermore, to support the theoretical results discussed above, we experimentally investigated the reflectance properties of a 10-layer SBR air-PLA with a Bragg frequency of $10 \; \mathrm{GHz}$ and a core radius of ${r}_{core}={\lambda }_{B}/2{n}_{air}$. It represents the portion of the reflected electromagnetic wave energy relative to the total energy of the incident electromagnetic wave. The reflectance measurements were made at different points along the core radius from 10 mm (near the inner PLA layer) to 1 mm (around the core center), and the results are shown in Fig. 4c. As expected, an omnidirectional stopband was obtained due to the constructive interference of the reflected waves at each boundary of the spherical shell. However, we observed that when the antenna was near the center, the maximum reflectance was less than one due to the transmission of electromagnetic energy to the outermost layers and the surrounding medium.

In addition to the omnidirectional stopband, we observed eigenmodes in the SBR. Figure S1b and c showed the calculated poles and zeros of the SBR scattering matrix, indicating one defect mode inside the cavity band gap and another eigenmode at the edge of the band gap. We measured the reflectance intensity at the eigenfrequencies $f=9.55$ GHz and $f=11.35 \;  \mathrm{GHz}$ at different points along the radius of the SBR core, as shown in Fig. 4d. Interestingly, we observed peaks in the reflectance intensity at these frequencies, which corresponded to the modes observed in the electromagnetic energy map. Furthermore, we observed that the reflectance intensity tended to increase as we moved away from the center of the SBR core. This trend was more noticeable at the frequency of $f=11.35 \; \mathrm{GHz}$, which is consistent with the behavior of the decay rate observed before. This behavior may be because this mode is mostly located in the core of the SBR close to the PLA layer.

### Theoretical and experimental scattering

**Figure 5:**
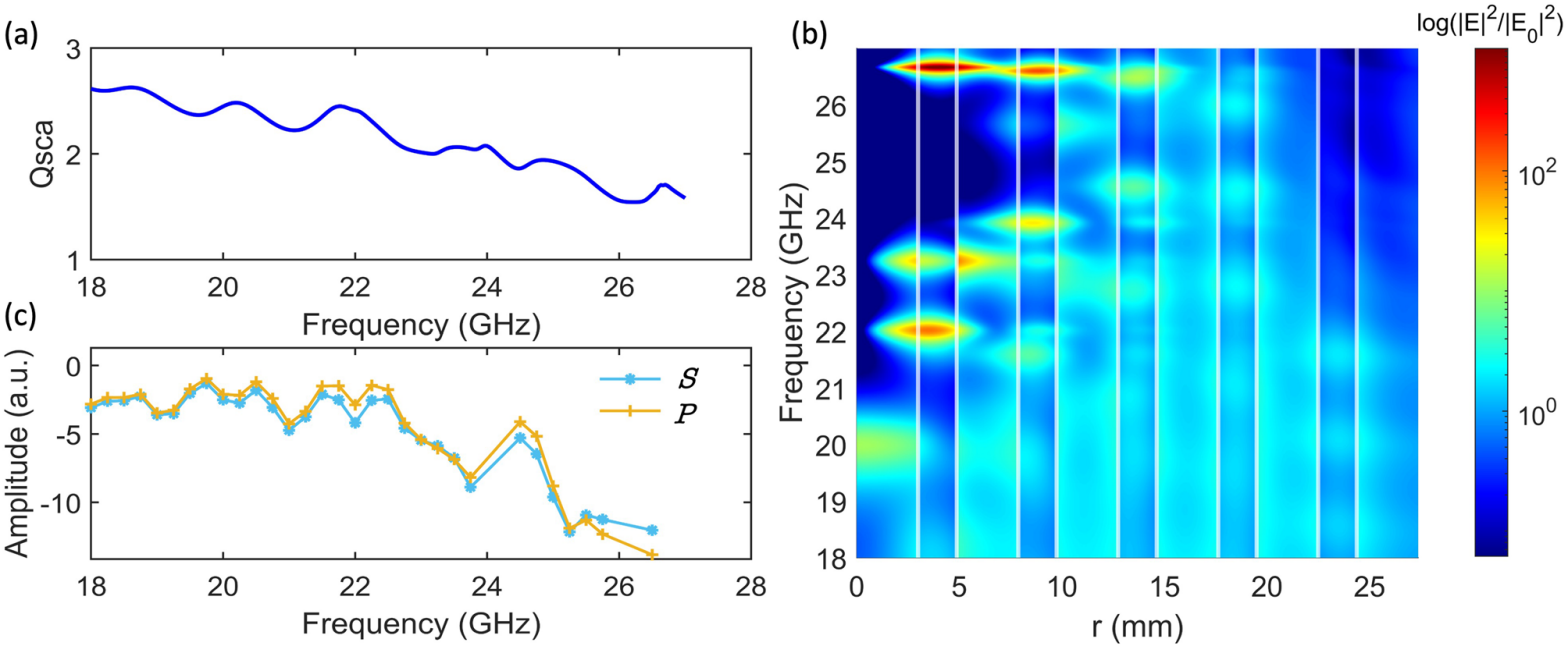
(**a**) Calculated total scattering efficiencies ${\mathrm{Q}}_{sca}$ for a 10-layer SBR up to order twelve of the multipole electric and magnetic contributions. (**b**) Spatial distribution of the normalized electric field intensities of a 10-layer SBR with ${r}_{core}={\lambda }_{B}/4{n}_{air}$ as a function of the frequency, the PLA layers are represented by white lines. (**c**) Scattering measurements of a 10-layer SBR for the $S$ and $P$ polarization.

This study uses theoretical calculations and experimental measurements to investigate the resonant modes of a 10-layer SBR with a core radius ${r}_{core}={\lambda }_{B}/4{n}_{air}$. Theoretical calculations based on T-matrix coefficients show that the SBR exhibits resonant modes of the whispering gallery type, reflected in the calculated total scattering efficiencies (${\mathrm{Q}}_{sca}$) shown in Fig. 5a. The peaks in the ${\mathrm{Q}}_{sca}$ correspond to the resonant modes of the SBR and provide valuable information about its electromagnetic properties.

To further investigate the resonant modes, we analyze the spatial distribution of the electric field intensities as a function of frequency, as shown in Fig. 5b. The results show that the electric field intensities are maximal at the resonant modes’ frequency. In particular, three maxima can be identified in the outermost layer of the SBR, corresponding to resonant modes of the whispering gallery type. These results agree with the calculated scattering efficiencies.

In order to validate the theoretical calculations, we perform experimental measurements of the scattering from the SBR using far-field monostatic measurements. The results, shown in Fig. 5c, confirm the presence of the resonant modes and the corresponding maxima in the scattering measurements. The SBR was excited by a $P$-polarized (parallel) and $S$-polarized (perpendicular) plane wave, and the results were similar in both cases, indicating the spherical symmetry of the SBR. The study of whispering gallery modes in the outermost layers of the SBR has important implications for potential applications in photonics and electronics. The ability to propagate electromagnetic energy through adjacent SBRs could be used to buffer or delay electromagnetic pulses more efficiently than with more conventional coupled resonators.

This work provides valuable insights into the electromagnetic properties of SBRs. The SBR can be scaled from nanometers to millimeters simply by changing the absolute dimension, making it possible to use it as a resonant cavity across the electromagnetic spectrum, particularly at optical, microwave, and, potentially, acoustic frequencies. In a broader sense, the concentric shells can be ellipsoids or any other arbitrary geometric structure.

The first importance of an SBR is achieving a complete photonic crystal property with a $4\pi$ full bandgap, which is challenging to attain in Cartesian systems without significant refractive index contrast. In contrast, the $k$-vector varies with direction in Cartesian coordinates, causing dispersion. However, in an SBR, a point source at the center results in all radiation being incident normally on the concentrical spheroidal surfaces, preventing the directional change of the $k$-vector and suppressing the emission radiation, thus increasing the cavity’s radiative lifetime with a high-quality factor. An infinite range of structures with various characteristics can be produced through the creation of local defects or the addition of specific materials like gases and nonlinear or active materials, allowing the implementation of directional loss or disorder. As a result, SBR lasers, masers, or “Rfasers” with true omnidirectional emission could be developed as we previously demonstrated.

## Conclusions

This paper comprehensively investigates millimeter-sized Spherical Bragg Resonators (SBRs) using 3D printing technology. The frequency dependencies of the reflection and transmission coefficients and the density of photonic states in the SBRs were examined through experimental and numerical analysis. Additionally, the influence of the source position on the resonator’s electromagnetic wave energy was investigated. The study also determined the scattering efficiencies of the multipole electric and magnetic contributions of a 10-layer SBR, and scattering measurements were taken for parallel and perpendicular polarizations. The results indicate that SBRs can significantly alter the density of electromagnetic states compared to free space, making them useful for enhancing or inhibiting spontaneous emission processes. The potential applications of SBRs include dielectric nanoantennas and optoelectronics devices across the entire electromagnetic spectrum. Overall, this work offers valuable insights into the development of new and innovative technologies based on SBRs, and paves the way for future research in this field.

## Methods

The theoretical analysis of the SBRs studied in this work was performed with a fast transfer-matrix method developed by Moroz et al.^19^. The feasibility of this fast and versatile method to study plasmonic and dielectric multilayered spherical nanostructures has been widely demonstrated. From the $T$-matrix, the scattering efficiency ${\mathrm{Q}}_{sca}$ at each multipole *l* and diagonal component *p* was obtained from the following expression^19,23^:

2 $${\mathrm{Q}}_{sca}=\frac{1}{{\left(rk\right)}^{2}}\sum_{p,l}\left(2l+1\right){\left|{T}_{pl}\right|}^{2}$$

where $r$ is the radius of the SBR and $k$ is the wave vector.

Also, through the Weierstrass factorization^57^ of each transverse electric ($TE$) and transverse magnetic ($TM$) component of the $S$-matrix ($\overline{\overline{S}}\equiv \overline{\overline{I}}+2\overline{\overline{T}}$, where $\overline{\overline{I}}$ is the identity matrix) is possible to obtain the eigenvalues of the SBRs as we depicted in^27^.

The electric field was calculated by the recursive transfer method, as is well described in^24^. While the normalized decay rate of a dipole is given by:

3 $${\gamma }_{\perp }=\frac{3}{2{x}_{d}^{4}}\frac{{n}_{d}}{{n}_{h}}\sum_{l}l\left(l+1\right)\left(2l+1\right){\left|{f}_{El}\left({x}_{d}\right)\right|}^{2}$$

4 $${\gamma }_{\parallel }=\frac{3}{4{x}_{d}^{4}}\frac{{n}_{d}}{{n}_{h}}\sum_{l}\left(2l+1\right)\left[{\left|{f}_{Ml}\left({x}_{d}\right)\right|}^{2}+{\left|{f}_{El}{\prime}\left({x}_{d}\right)\right|}^{2}\right]$$

The symbols “⊥” and “∥” refer to the dipole’s perpendicular and parallel orientations (with respect to the *z*-axis) at position *d*, respectively. The prime symbol denotes differentiation with respect to the argument. Additionally, ${n}_{h}$ denotes the refractive index of the host medium, $l$ represents the angular quantum number, and ${f}_{\mathrm{El},\mathrm{Ml}}\left({\mathrm{x}}_{\mathrm{d}}\right)$ represents linear combinations of Riccati-Bessel functions. The dimensionless size parameter ${\mathrm{x}}_{\mathrm{d}}$ is defined as ${\mathrm{x}}_{\mathrm{d}}={k}_{\mathrm{d}}{r}_{\mathrm{d}}$ where ${k}_{\mathrm{d}}=2\pi {n}_{\mathrm{d}}/\lambda$.

### SBR fabrication and experimental measurements

**Figure 6:**
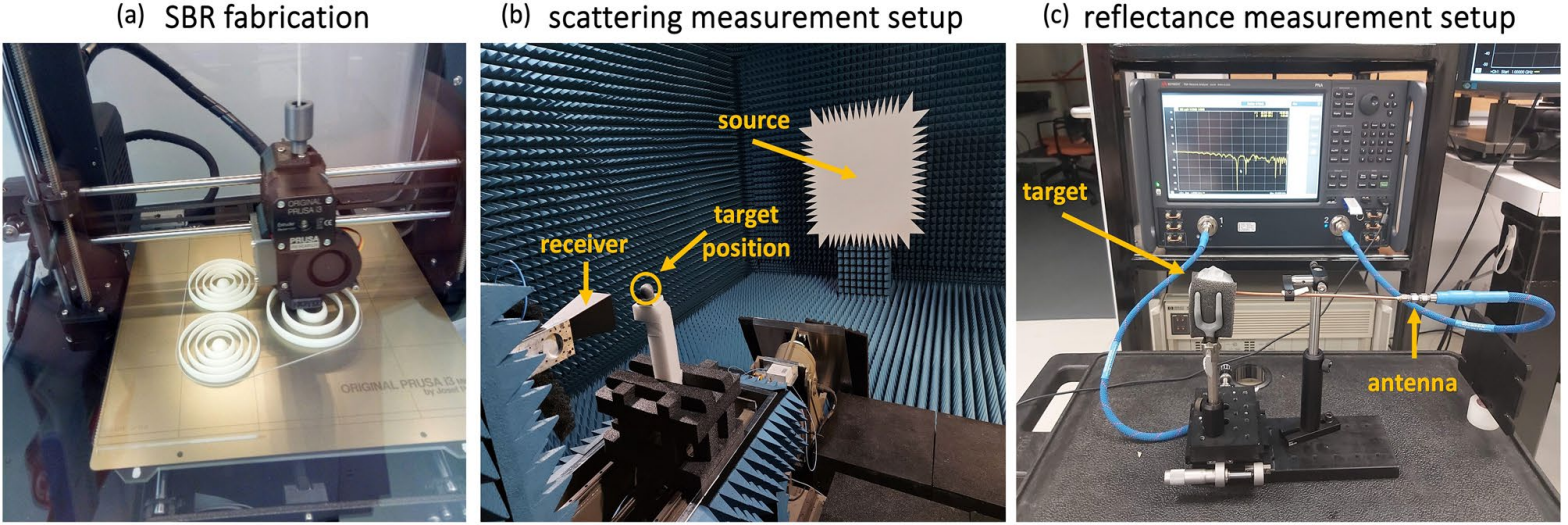
(**a**) SBR fabrication by 3D printing in PLA. Different sizes of 3D-printed. (**b**) Scattering and (**c**) reflectance measurement setup.

Figure 6a shows the SBRs fabrication process using Value PLA-polylactide (PLA)^47^ via 3D printing using an Original Prusa i3 MK3 with a resolution of $0.05 mm$. PLA is characterized by a dielectric constant around $2.75\pm 0.05$ and loss tangent $\left(1.1\pm 0.2\right)\times {10}^{-2}$ in the microwave range^58^. The process involved depositing a thin rod followed by the outermost semi-spheroid shell, then extending the rod and adding the next inner semi-spheroid. In this study, an air gap was used to imitate the neighboring semi-spheroidal shell, but another material could be deposited to alter the refractive index. The thicknesses of the air and PLA shells were equivalent to a quarter of the optical length ${\lambda }_{B}/4{n}_{air}$, where ${\lambda }_{B}$ represents the center of the stop band and *n* is the refractive index of the air or PLA. As a result, the dimensions and periodicity of the concentric spheres determined the resonance frequencies. The process was repeated until all semi-spheroidal shells were deposited, creating the innermost spheroid and outermost shell, resulting in the final SBR. This study considers SBRs 3D printed with core radiuses of ${\lambda }_{B}/4$ and ${\lambda }_{B}/2$, using five and three concentric spherical dielectric PLA layers spaced by air. The center of the stop band was fixed at $10 \; \mathrm{GHz}$ and $25 \; \mathrm{GHz}$.

Keysight's network analyzer N5224B was used to measure the antennas' reflected and transmitted voltage signal. The operating frequencies are from $10 \; \mathrm{MHz}$ to $43.50 \; \mathrm{GHz}$. Port 1 was connected to a coax cable PE-118SR through an SMA-K connector to lead the signal up to the center of the SBR. Measured $\left|{S}_{11}\right|$ parameters of SBRs with a different number of layers (${n}_{l}=10$ and ${n}_{l}=6$) are shown in Fig. S3. In addition, the SBR was mounted on a mobile station to adjust the antenna's position with respect to its center, as shown in Fig. 6c. With this configuration, we obtained the magnitude of the reflected voltage signal ${\left|{S}_{11}\right|}^{2}$. Port 2 was connected to a horn antenna for the transmission measurements to estimate the magnitude of transmitted signal ${\left|{S}_{21}\right|}^{2}$ close to the SBR surface.

To extract the scattered electric field of the SBR from $18$ to $26 \; \mathrm{GHz}$, we conducted far-field monostatic measurements in an anechoic chamber. Figure 6b illustrates our configuration within the anechoic chamber. The SBR under test was positioned on a post, and a plane wave illuminated the target. The setup enables us to measure the amplitude of the scattered electric field. Our measurements covered both polarizations, $S$ and $P$. The $S$ polarization corresponds to the electric field being perpendicular to the plane containing the SBR, source, and receiver. In contrast, the $P$ polarization corresponds to the case where the electric field is parallel. To switch between the two polarizations, the source, and receiver were mechanically rotated by 90°. We utilized a robust polyurethane foam as the base material to minimize absorption and achieve the lowest possible refractive index^59^.

### Supplementary Information

Supplementary Figures.

## Data Availability

Data underlying the results presented in this paper are not publicly available at this time but may be obtained from the corresponding author upon reasonable request.
