# Supplementary material for: Experimental characterization of Spherical Bragg Resonators for electromagnetic emission engineering at microwave frequencies
Source: Sci Rep. 2023 Nov 22;13:20532. doi: 10.1038/s41598-023-47059-y (PMC10665359; doi:10.1038/s41598-023-47059-y)
Supplement: Supplementary file 1 — Supplementary Figures. [file 41598_2023_47059_MOESM1_ESM.docx]

**Supplementary Information**

Experimental characterization of Spherical Bragg Resonators for electromagnetic emission engineering at microwave frequencies

Yalina García-Puente,^1,*^ Jean-Jacques Laurin,^2^ and Raman Kashyap^1,2^

^1^Department of Engineering Physics,

^2^Department of Electrical Engineering, Poly-Grames Research Centre, Polytechnique Montreal, 2900 Édouard-

Montpetit, Montreal, Qc H3T 1J4, Canada

* yalina-2.garcia-puente@polymtl.ca


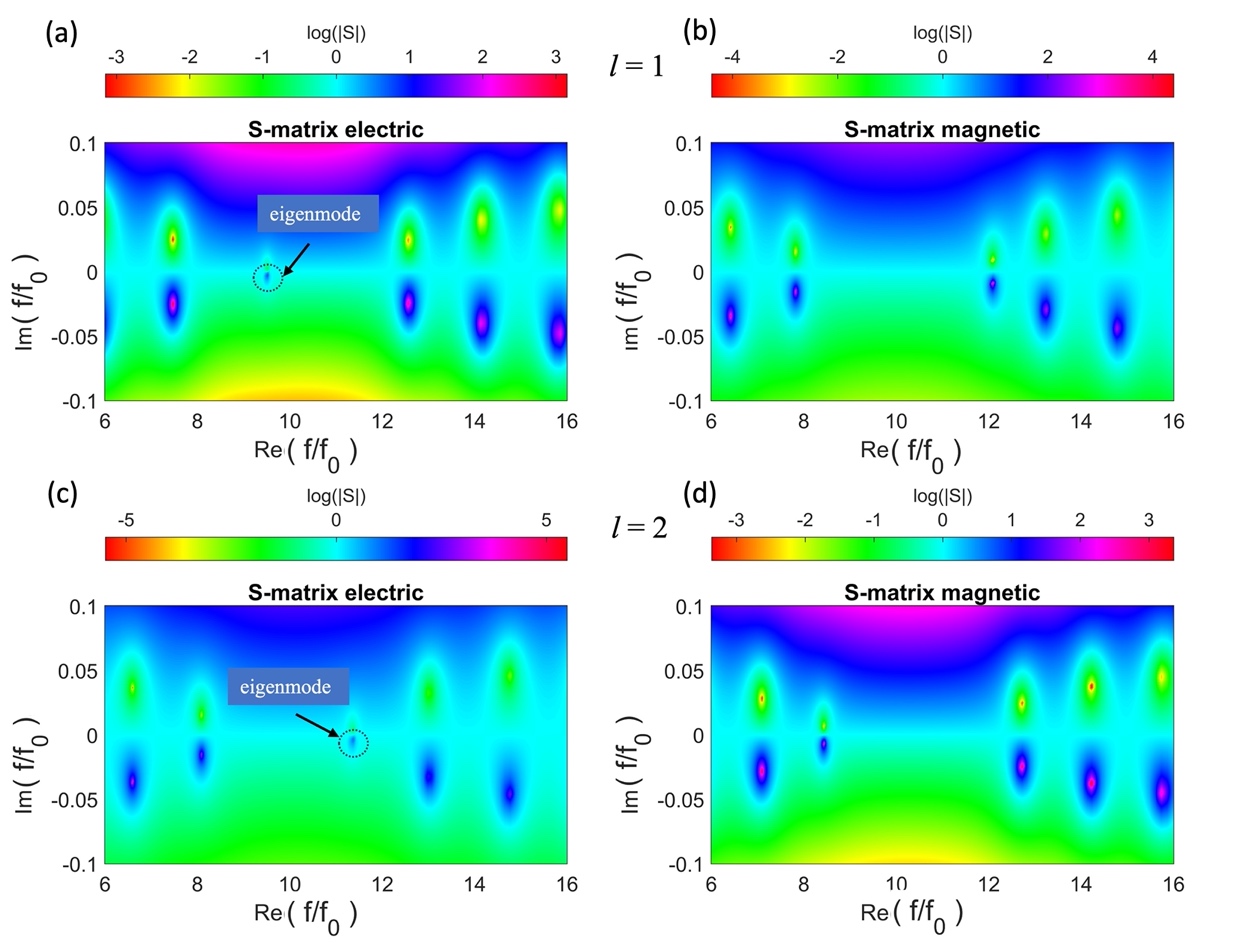


**Figure S1.** Calculated maps of the $S_{pl}$ matrix component in the complex frequency plane for **(a)** TE1, **(b)** TM1, **(c)** TE2 and **(d)** TM2 in 10-layer SBR air-PLA with a Bragg frequency of $10 GHz$ and a core radius of $r_{core}={\lambda_{B}}/{2n_{air}}$.


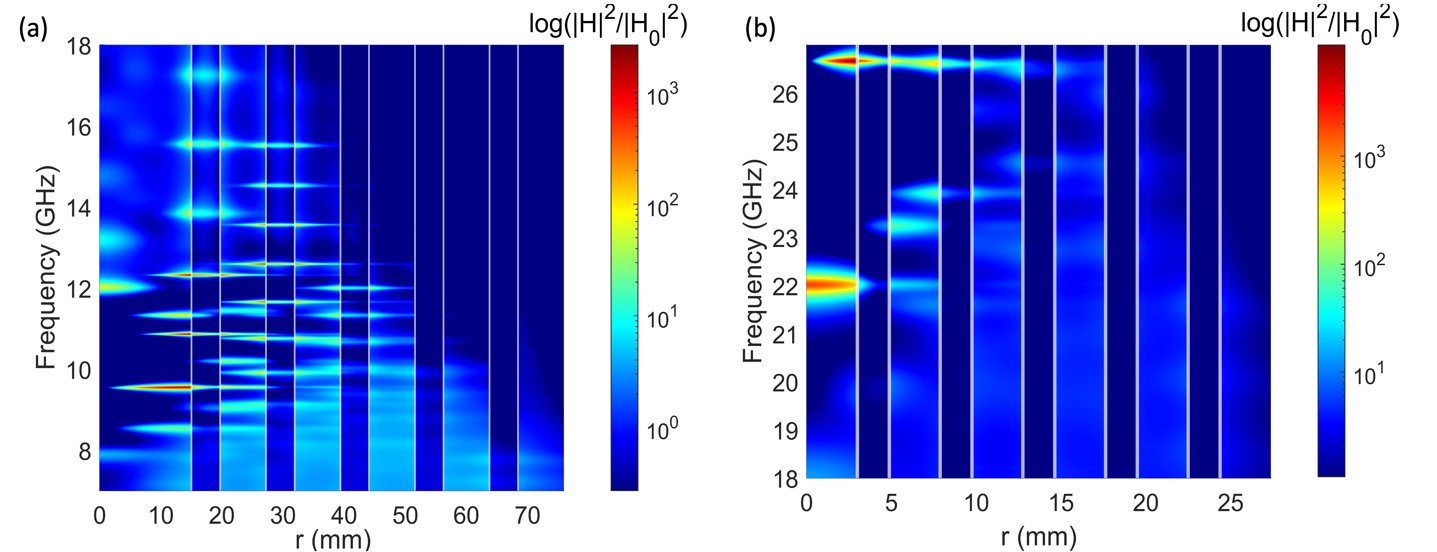


**Figure S2.** Spatial distribution of the normalized magnetic field intensities of a 10-layer SBR with **(a)** $r_{core}={\lambda_{B}}/{2n_{air}}$ and **(b)** $r_{core}={\lambda_{B}}/4n_{air}$ as a function of the frequency, the PLA layers are represented by white lines.


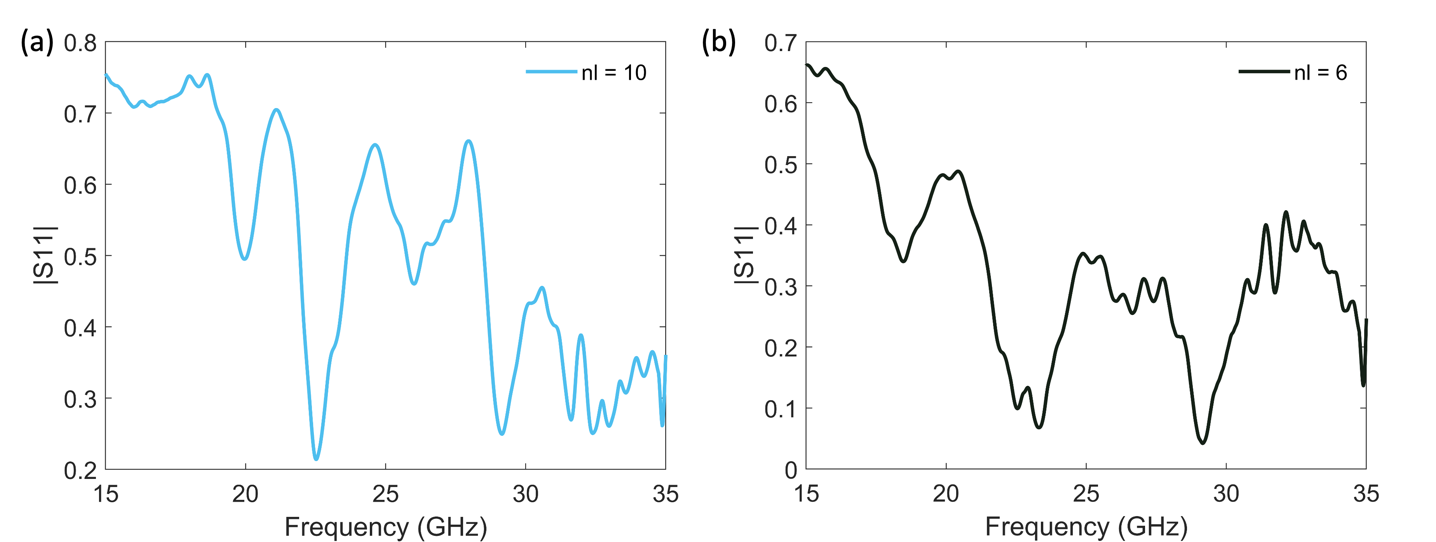


**Figure S3.** Measured $\left| S_{11} \right|$ parameters of SBRs with a different number of layers, **(a)** $n_{l}=10$ and **(d)** $n_{l}=6$, at the core of the structure.
